# Retraction: Agile soft template array fabrication of one-dimensional (1D) polyaniline nanocomposite fibers for hydrogen storage

**DOI:** 10.1039/d5ra90140e

**Published:** 2025-11-20

**Authors:** Ameena Parveen, S. Manjunatha, M. Madesh Kumar, Aashis S. Roy

**Affiliations:** a Department of Physics, Government Degree College Yadgir-585201 Karnataka India; b Department of Physics, SSA Government First Grade College Ballari-583101 India; c Department of Physics, REVA University Bangalore-560064 Karnataka India; d Department of Chemistry, S. S. Tegnoor Degree College Gubbi Colony-585104 Karnataka India aashisroy@gmail.com +91-9108809031; e Department of Industrial Chemistry, Addis Ababa Science and Technology University Addis Ababa 500013 Ethiopia

## Abstract

Retraction of ‘Agile soft template array fabrication of one-dimensional (1D) polyaniline nanocomposite fibers for hydrogen storage’ by Ameena Parveen *et al.*, *RSC Adv.*, 2024, **14**, 25347–25358, https://doi.org/10.1039/D4RA04710A.

The Royal Society of Chemistry, with the agreement of the named authors, hereby wholly retracts this *RSC Advances* article due to concerns with the reliability of the data.

Concerns were raised regarding the XRD data in Fig. 3 and the SEM images in Fig. 4, as this contains a rotated duplicated image panel. The authors were asked to provide the raw data and an explanation. The XRD data provided by the authors does not match the data in the paper. The authors admitted that the SEM images in panels 3c and f were duplicates, and asked for a correction for Fig. 3f; however, the raw data contained file names that did not match the labelling from the paper, and the replacement image appears to have been collected under different settings to the other images. An independent expert has reviewed the raw data and response and deemed them unsatisfactory.

Given the significance of these concerns, the Editor has lost confidence that the findings presented in this paper are reliable.

The authors were informed about the retraction of the article. Ameena Paerveen, Aashis S. Roy and M. Madesh Kumar have agreed with the decision. The other author has not responded.

Signed: Ameena Paerveen, Aashis S. Roy and M. Madesh Kumar

Date: 13^th^ November 2025

Retraction endorsed by Laura Fisher, Executive Editor, *RSC Advances*

